# Trust and The Acquisition and Use of Public Health Information

**DOI:** 10.1007/s10728-021-00436-y

**Published:** 2021-11-09

**Authors:** Stephen Holland, Jamie Cawthra, Tamara Schloemer, Peter Schröder-Bäck

**Affiliations:** 1 Departments of Health Sciences and Philosophy, University of York, York, YO10 5DD UK; 2 Department of Philosophy, University of York, York, UK; 3 Department of International Health, Care and Public Health Research Institute (CAPHRI), Maastricht University, Maastricht, Netherlands; 4 Department of International Health, Care and Public Health Research Institute (CAPHRI), Maastricht University, Maastricht, Netherlands

**Keywords:** Health data management, Public health information, Trust, Privacy, Value congruence

## Abstract

Information is clearly vital to public health, but the acquisition and use of public health data elicit serious privacy concerns. One strategy for navigating this dilemma is to build 'trust' in institutions responsible for health information, thereby reducing privacy concerns and increasing willingness to contribute personal data. This strategy, as currently presented in public health literature, has serious shortcomings. But it can be augmented by appealing to the philosophical analysis of the concept of trust. Philosophers distinguish trust and trustworthiness from cognate attitudes, such as confident reliance. Central to this is value congruence: trust is grounded in the perception of shared values. So, the way to build trust in institutions responsible for health data is for those institutions to develop and display values shared by the public. We defend this approach from objections, such as that trust is an interpersonal attitude inappropriate to the way people relate to organisations. The paper then moves on to the practical application of our strategy. Trust and trustworthiness can reduce privacy concerns and increase willingness to share health data, notably, in the context of internal and external threats to data privacy. We end by appealing for the sort of empirical work our proposal requires.

## Introduction

This paper addresses the problem that information is vital to public health, but the acquisition and use of health data elicit serious privacy concerns. The paper focuses on the response that building ‘trust’ in institutions responsible for health data reduces people’s privacy concerns and increases willingness to disclose their information. The first section explains the problematic, and shortcomings of current versions of the trust-based response. Sections 2–4 present our solution, which augments current strategies for building trust by appealing to the philosophical analysis of the concept of trust. Section 5 demonstrates that objections to this ‘augmentation strategy’ can be met by more nuanced accounts of our proposal. Section 6 discusses practical applications of our suggestion; Section 7 describes the sort of further empirical work our proposal requires.

## Privacy, Utility and Building ‘Trust’

That information is vital to public health has been brought home by the Covid-19 pandemic occurring at the time of preparing this article. All measures to deal with this coronavirus outbreak require information, ranging from epidemiological data on infection rates and local outbreaks, to virology data to develop a clinical response. But the Covid-19 crisis is only a particularly acute case in point, because all public health endeavours—including all health prevention and promotion activities—are based on gathering and using information.^1^

Gathering and using public health data can create a dilemma.^2^ One the one hand, the benefits of acquiring and processing health information are as great as the benefits of public health, since the latter depends on the former. On the other hand, many health data are personal and sensitive, so gathering and using such data generates privacy concerns. This dilemma is again readily illustrated by the current Covid-19 crisis. In the UK at the time of writing, for example, contact tracing is crucial to managing the outbreak, but the UK Government’s ‘track and trace’ system has generated substantial informational privacy concerns [4]. More generally, it has recently been argued that there is a discernible tendency in responses to this utility-privacy dilemma to prioritise privacy protection at the expense of public health, so numerous ways of addressing the dilemma have been proposed in order to protect public health from this distinctive threat [11].

One such strategy centres on ‘trust’.^3^ The basic idea is that the more people ‘trust’ institutions responsible for managing their health data, the less will be their privacy concern and, in turn, the greater their willingness to contribute personal information. As a result, there are frequent invitations and proposals in the public health literature to ‘build trust’ in organisations responsible for health data. Such calls to build ‘trust’ recommend the same strategy: reassure the public by identifying, establishing, and improving regulatory mechanisms imposed on relevant institutions.^4^ Versions of this strategy include eliciting barriers to providing personal data in order to identify appropriate regulatory changes; guidelines for collecting, storing and using data; and frameworks for refining health information technology systems [16]. These general attempts to reassure the public by increased oversight and greater accountability are augmented by tighter restrictions on specific types of public health data, including electronic health records and biobanks [18, 19]. Likewise, improved governance structures are recommended for specific uses of public health data, such as secondary analysis [26] and emergency preparedness [20].

But there are major shortcomings of attempting to build ‘trust’ by more stringent regulation. There are issues around both cost and effectiveness. Regarding costs, there are two main problems, both of which are particularly important in health care contexts. First, regulatory mechanisms are financially expensive, because regulation is achieved by ever more expensive information technologies and audit systems, all of which incur overheads in terms of staffing, utilities, and other operating costs. This is particularly significant given the scarcity of healthcare resources. Second, it has been argued that there is already a good deal of overregulation of health data, notably, in the context of health related research [11]. Building ‘trust’ by increasing regulation will worsen this ‘regulation inflation’, incurring the opportunity cost of foregoing beneficial research data and other information.

Regarding effectiveness, increased regulation is ineffectual in two main ways. First, the strategy tends not to reduce, but merely redirect, public mistrust. Specifically, public mistrust of health care institutions which manage health information is redirected towards institutions responsible for implementing and monitoring the proposed regulatory innovations [22]. The second, even bigger problem is that the strategy is bound to fall short, because increasingly complex IT systems and ever more sophisticated ‘adversaries’ (i.e., those intent on re-identifying data) create an unpredictable and unmanageable array of potential data breaches. As a result, regulators are inevitably playing catch-up, as evidenced by well publicised information leakages, so increased regulation does not satisfy the public’s need for reassurance.

Given all these shortcomings, attempts to meet the utility-privacy dilemma by building ‘trust’ seem hopeless. But there is an alternative way of appealing to ‘trust’ to help address the privacy-utility dilemma in public health, which is gaining some traction [11, 21]. This starts with the fact that calls to build ‘trust’ in institutions responsible for managing public health data, of the sort just sketched, do not provide an analysis or definition of trust but, rather, take the concept as read. Specifically, in the relevant public health literature, ‘trust’ is used in a univocal and colloquial sense to capture the general idea of confidently relying on institutions managing health information. But trust is the subject of conceptual analysis in a number of disciplines; might some insights from analyses of trust help revive the trust-based response to the utility-privacy dilemma in public health?

## The Concept of Trust

The philosophical literature on trust is an obvious place to start, since it provides the most substantial and sustained conceptual analysis.^5^ Philosophical analyses of trust are contested and principally focused on epistemology, specifically, the epistemic status of beliefs formed on the basis of testimony. Nonetheless, there is a fairly straightforward, well documented and uncontentious insight in this literature.

Philosophers think of trust as one of the interpersonal attitudes required when a person is dependant on another to achieve some goal under conditions of uncertainty. Standard examples include intimate relationships (spouses and other partners depend on one another), forming beliefs on the basis of testimony (we depend on others to provide true information), and practical goals (we depend on others to do jobs that are beyond us). Trust is an appropriate term to describe all such cases: I trust my partner not to have an affair; I trust the person on the street to give me accurate directions; I trust the dentist to fix my teeth. The relevant philosophical insight is that these uses of the single term, ‘trust’, obscures the fact that two distinct interpersonal attitudes are appropriate in these various sorts of interactions.

One way of putting this insight is that ‘trust’ is ambiguous, but it is clearer—and has become established—to use two referring terms for each of the relevant attitudes, respectively. So, one attitude is usually called reliance, whilst ‘trust’ is reserved for the other. In the examples just cited, one relies on a passer-by to provide true information when asked for directions, or on a dentist to do a good job, but the attitude life-partners have to one another is different, because their relationship has gone beyond mere reliance to become one of trust. Each of these attitudes has a correlative, namely, reliability and trustworthiness, respectively: A relies on B because A thinks B is reliable; by contrast, A trusts B because A thinks B is trustworthy. This distinction between reliance and trust needs to be clarified sufficiently to pursue our argument, but it is so familiar from the philosophical literature that it can be sketched fairly quickly here.

The main way trust and reliance are distinguished is by certain reactive attitudes appropriate to failures of trust but not reliance.^6^ Specifically, trusting makes one vulnerable to a sense of betrayal and feelings of resentment. Failures of trust—finding out one’s partner is having an affair, for example—are hurtful, elicit strong emotions and cause psychological harm. By contrast, when someone on whom one confidently relied turns out to be unreliable, the upshot is disappointment which, though clearly important and often strong, has none of the visceral quality of betrayal. This is a difference in phenomenology, not in the magnitude of the overall effect: misplaced reliance can be practically disastrous, and some failures of trust turn out for the best in the long run; nonetheless, the two feel very differently to one another and, typically, misplaced trust is not only a distinctive but also a far worse experience.

Although this describes the trust/reliance distinction, it doesn’t explain it. In the philosophical analysis, the distinction is typically explained by motives and values. What makes a trusted party trustworthy is that they are motivated by a concern to act in the trusting party’s best interests. This is captured in various ways, such as that we trust people when we believe they will act in good faith and with our interests at heart [15]. Regarding values, trust is appropriate when the trusting and trusted parties recognise and share one another’s values. This is referred to as value congruence [2, 14]. To illustrate these features, A trusts their partner B not to have an affair, because A knows that B is motivated to act in A’s best interests, and A and B have shared values regarding such matters.

These motives and values explain the reactive attitudes distinctive of trust. Failures of trust elicit a sense of betrayal and feelings of resentment, and are typically so shocking and hurtful, because they reveal that the trusted party either was not motivated to act in the trusting party’s best interests, or did not share the trusting party’s values, or both. By contrast, reliance is calculative—i.e., it is based on A’s rational appraisal of the likelihood that B will behave a certain way—as opposed to being based on perceived motives and values. For example, A’s reliance is determined by their calculation as to which passer-by is most likely to provide good directions, or which dentist is most likely to do a good job. Hence, misplaced reliance might well make A feel silly for having miscalculated B’s reliability, angry at being let down, and so on; but this is mere disappointment, however strongly felt (and practically important), as opposed to the distinctively hurtful experience of failures of trust.^7^

## Trust, Reliance, and The Public Health Literature

A major motivation for this paper is that the philosophical distinction between trust and reliance just sketched is entirely lost in public health literature. Specifically, in the calls to build ‘trust’ in public health institutions described in the previous section, terms such as trust, reliance, confidence (and cognates) are used interchangeably. In fact, all such terms are used to refer to what philosophers would recognise as reliance (as opposed to trust). This is clear from specific strategies for building ‘trust’ in public health institutions that were sketched above. To recall, barriers and willingness to contribute data are elicited in order to determine how best to persuade people that institutions responsible for managing their health data are reliable. Guidelines and frameworks are proposed in order to guarantee the reliability of health data management and, in turn, encourage public confidence. Likewise, the point of regulatory mechanisms such as increased oversight, greater transparency, and increased accountability—including restrictions on specific types and uses of public health information—is to increase public confidence in organisations responsible for their health data by reassuring them that they are reliable.^8^

So, every aspect of the strategy to build ‘trust’ espoused in the public health literature is in fact designed to ensure that public health institutions are reliable in order to encourage more confident reliance. And, as explained above, this strategy has serious shortcomings, around both cost and effectiveness. But the philosophical analysis reveals a cognate attitude: not mere reliance based on reliability, but trust based on trustworthiness. And this is the basis of the proposal that there is an alternative trust-based strategy for addressing the dilemma between the public utility of health data and individuals’ informational privacy concerns. Specifically, the alternative strategy is to appeal to public trust (not mere reliance) in institutions responsible for managing health data, on the basis of the latter’s trustworthiness (not mere reliability).^9^

But what, precisely speaking, is this alternative strategy? After all, there are a number of possible ways of combining the current strategy of gaining public confidence by improving regulatory mechanisms, with fostering public trust in trustworthy institutions. So, exactly how is the appeal to trust and trustworthiness supposed to go? In this paper, we focus on what we take to be the most plausible answer mooted in the literature [11 at pp. 111–129].

## Reliance, Trust, and Value Congruence

The appeal to trust we focus on retains attempts to build public confidence by better regulation, but augments these by also displaying institutions’ trustworthiness to elicit public trust. This ‘augmentation strategy’ is intended to address the utility-privacy dilemma and thereby increase willingness to share personal health data for public health purposes. The point of this paper is to critique this alternative trust-based strategy, at the point at which it is gaining some traction [21].^10^ But first, we need a more detailed account of the strategy. Precisely how is reliance/reliability to be augmented by trust/trustworthiness? What are the details of this strategy?

To recall, the distinction between trust and reliance was explained in the philosophical analysis by motives and values: people trust when they see the trusted party as trustworthy, as based on the former’s perception of the latter’s motivations and values. At this point, a brief theoretical clarification is important to our argument. There is much debate in the philosophical literature as to which of rival candidates—not only values and motives, but others, such as intentions, obligations, and so on—is the defining characteristic of trust/trustworthiness. But we think much of this is unnecessary, because this is a pseudo-dispute, as opposed to a substantive dispute. To illustrate, take a debate over whether the crucial difference between trust and reliance is motives or values, in which it is claimed that motive is what really matters; in other words, the crucial component of the trusting relationship is that the trusting party (A) thinks the trusted party (B) is motivated to act with their (A’s) best interests at heart. We don’t see this as a genuine dispute, because motives are not really a rival to value congruence. After all, why does B have A’s best interests at heart? Presumably, because B values A and A’s wellbeing. But so does A (i.e., A also values A and A’s wellbeing). So, what look like rival candidates for the defining characteristic of the trusting relationship collapse into one another. The same move can be made *mutatis mutandis* for any putative rival to value congruence as the crucial component of trust. At root, the rival will be about values, and trust is warranted by recognition of, and congruence with, those values.

On this basis, we pursue our argument in terms of value congruence: not because value congruence has won the theoretical dispute as to which is the crucial explanatory component of trust, but because ‘value congruence’ captures all candidates in that pseudo-dispute. So, now we can present our complete account of the trust-based response to the privacy/utility dilemma in public health. Calls to ‘build trust’ found in the public health literature really amount to increased regulation of institutions responsible for public health data in order to encourage more confident public reliance. We propose that this is augmented by fostering trust in those institutions on the basis of their perceived trustworthiness. And fostering trust amounts to institutions developing, displaying, and abiding by values which are congruent with those of the public.^11^ This will alleviate the public’s informational privacy concerns and thereby increase their willingness to contribute health data.^12^

## Meeting Objections

This section defends the augmentation strategy from seemingly strong initial challenges. Although this is not intended to be exhaustive, the discussion demonstrates that challenges can be met by a more nuanced account of the strategy.

An immediate response to the strategy under discussion is that trust is an interpersonal attitude, so it is irrelevant to the way people relate to organisations. In other words, people trust other people, on the basis of perceived trustworthiness, so it is a category error to think people trust institutions. This challenge is strengthened by the fact that the most obvious way of meeting it won’t work. This obvious solution is that people do not trust organisations per se; rather, they trust the individual professionals who staff them. For example, patients don’t trust hospitals, they trust individual doctors, nurses, administrative staff, etc., who work there and with whom they interact. This seems to meet the challenge by recasting public trust in organisations as interpersonal after all. But it clearly won’t help in the current context, because members of the public do not interact with public health professionals who staff public health institutions in the way they interact with their doctor and other health care providers.^13^

The better way to meet the challenge starts by clarifying it. To recall, the two main components of the philosophical analysis of trust are reactive attitudes to failures of trust (principally, a sense of betrayal and feelings of resentment) and value congruence (i.e., trust is elicited by perceiving trustworthiness on the basis of shared values). So, the objection under discussion is that these reactive attitudes, and value congruence, do not belong in the relationship between people and institutions. Put like this, the objection is simply false, because there is a wealth of theoretical and empirical support for the view that people do trust organisations. For example, this is well established in sociological critiques of organisations [24]. And analysts in other disciplines have defended the notion of public trust, showing that people not only rely on, but also trust, organisations as well as other people; that failures of trust in organisations elicit the relevant reactive attitudes, and that trust is based on the perception that the organisations in question shares the public’s values [25].

The challenge can be revived by arguing that, although the public trust some sorts of organisations, they don’t trust those relevant in this context, namely, health care institutions or institutions responsible for managing data. But this is also unconvincing. There is clear anecdotal evidence of value congruence between the public and healthcare institutions; a case in point is the British public’s response to the current coronavirus pandemic, which is characterised by a recognition that the NHS shares its values, resulting in an outpouring of support.^14^ The very nature of public health institutions suggests that their values will align with the public’s, since the point of public health organisations is to act in the public’s best interests. And this is confirmed by empirical evidence that there is public trust in healthcare actors, grounded in value congruence [14], and that trust based on shared values reduces privacy concerns and thereby increases willingness to share personal information [2]. This has been transposed to the management of health data; for example, as previously mentioned, Sheehan et al.’s discussion of trust and trustworthiness in the context of using patient data for research in the NHS alludes throughout to institutions’ values and their congruence with the public [21].^15^

Still, the objection isn’t fully dispelled, because it does seem odd to think that people trust organisations in exactly the same way as they trust each other. For example, it would be odd for someone to feel as betrayed by, or as resentful towards, an organisation as they would when let down by a partner or best friend. But this version of the challenge can be met by a more nuanced account of the trusting relationship. So far, we have starkly contrasted reliance and trust for the sake of clarity and brevity, but the taxonomy of relevant attitudes is more complex than this. To illustrate, in this journal, Holland and Stocks [12] distinguished two ‘species of trust’: ‘general trust’ exists between partners, close friends, and so on; ‘specific trust’ is an attitude one person adopts towards another in order to achieve a goal, but their relationship has developed beyond mere reliance.^16^ The relevant reactive attitudes—such as feeling betrayed and resentful—are appropriate to both species of trust, but to a different degree. For example, betrayal of general trust ‘is profound and shocking, causing intense psychological harm’ [12 at p. 271]; by contrast, failures of specific trust also generate a sense of betrayal and feelings of resentment, but to a lesser extent and with a different qualitative feel.

Holland and Stocks [12] illustrate their general/specific trust distinction by contrasting the reliance one places in a taxi service, with the trust (in the ‘specific’ sense) a passenger gradually develops in their regular taxi driver whom they get know over a period of time. It would be inappropriate for the passenger who trusts their taxi driver (in the ‘specific’ sense) to experience ‘profound and shocking betrayal’ or ‘intense psychological harm’, if they let them down. Nonetheless, they would feel betrayed, and resentment is appropriate, albeit to a lesser and amended extent.^17^ Assuming the attitude appropriate to organisations is that of ‘specific trust’ deals with the challenge under discussion: we have the reactive attitudes distinctive of trust—for example, feeling betrayed and resentful—towards organisations we find trustworthy, but in an attenuated sense and to a lesser degree than in the case of ‘general trust’.^18^

But these sorts of worries have still not been fully dispelled. There is another version of the challenge that, although trust and trustworthiness are appropriate to organisations as well as in interpersonal relations, this does not extend to the sort of institutions in which we are interested. This other version focuses on the fact that public health information is acquired and used by a network of state-backed government agents—including national, regional, and local bodies—and mistrust of government is well documented. More nuanced accounts of the trusting relationship, as illustrated by Holland and Stocks [12], cannot be enlisted here, because the challenge is that people do not trust governments at all, not even in an attenuated sense or to a lesser extent. And this challenge is strengthened further because it arises even in political systems where there is a democratic mandate: people in complex, developed democracies are too far removed from political decision making to feel reassured that they can trust government agents; and attempts to enhance democratic legitimation—opinion polls, citizens’ juries, and so on—fall short of providing the requisite reassurance to engender public trust grounded in the perceived trustworthiness of governments and their agents.

But this challenge is not fatal to the augmentation strategy. Distrust of government in the context of public health is not uniform. For example, public mistrust is most strongly elicited by liberty-limiting public health interventions which are paternalistically motivated—hence, paternalism is a major motivation for and feature of public health ethics [10]—but the public are much less distrustful of public health measures clearly aimed at avoiding third party harms (hence the generally high rates of compliance with current restrictions to reduce transmission of Covid-19). So, we need to ask how forceful this challenge is in the specific case of public trust in institutions responsible for managing public health information. And the answer is, ‘not very’, because the vast majority of public health work—and associated information management—is not the sort that elicits public distrust on the grounds of being state sanctioned. The overwhelming majority of people are not outraged by standard public health information gathering practices, or data collection during public health emergencies. So, we can acknowledge that the public mistrust governments and their agents—and even that the public has serious misgivings about the role of the state in public health interventions which flout individual liberties—whilst promoting public trust in public health information management.^19^

## Practical Application

To recap, the strategy under discussion is to augment reliance on institutions responsible for public health information, by eliciting trust in those institutions, based on perceived trustworthiness due to value congruence. This strategy has been motivated, clarified, and defended. The next question is about the practical significance of all this. After all, the point of the discussion is to address the utility-privacy dilemma in public health data management described at the outset; precisely how would eliciting public trust in trustworthy institutions help with this?

The central practical question is how reliance and trust are supposed to relate to one another. In the literature there is a consensus that this depends on the institution in question. The public relate to different organisations in various ways, so the interplay between reliance and trust will differ from institution to institution. For example, Townley and Garfield [25 at pp. 103ff.) refer to ‘public institutions of different kinds to which the public as a whole or individual members of a society bear very different kinds of relations … [hence] … there are no general rules governing where each is appropriate’. But things are a bit more straightforward in the present context, because we are focused on a specific sort of institution, namely, those responsible for public health data. Of course, the precise admixture of reliance and trust is still hard to discern, not least because of the size and complexity of the organisations in question. Nonetheless, there are some fairly clear themes when it comes to the practical question of the admixture of reliance and trust in the institutions we are focused on.

The way into this is to distinguish two sorts of informational privacy concerns, based on external versus internal threats. External threats are from adversaries intent on maliciously acquiring and using people’s information; internal threats are from failures within organisations processing personal data. Classic examples of the former include hackers gaining access to IT systems to acquire people’s information for commercial or other malicious purposes. Internal threats range from organisations being simply complacent or negligent about people’s information, to malicious intent, such as using or selling data in ways people are unaware of, for commercial or other reasons. This distinction helps to clarify the practical value of augmenting reliance with trust in the context of public health information.

Let’s focus first on external threats. The main response to adversaries with malicious intent—and the only way to alleviate the public’s informational privacy concerns about them—is more reliable data management. Trust and trustworthiness are not clearly relevant here.^20^ For example, the appropriate response to the external threat of persistent cyber attacks is better cyber security—including continually upgrading security software, monitoring data breaches, and so on—not displays of value congruence or any such like. Since the augmentation strategy clearly does not obviate the need for reliability, it will not avoid the costs associated with increased regulation. And, as argued above, these costs are considerable, because increasing reliability means devising, imposing, and refining regulatory mechanisms, which are expensive. This is a major limitation of the trust-based strategy, one that is underplayed in the burgeoning literature on this topic: the public will continue to require that organisations responsible for public health data are reliable, in order to meet informational privacy concerns based on external threats, no matter how much they trust those organisations on the basis of perceived trustworthiness due to shared values.

Things are more promising when we turn to informational privacy concerns based on internal threats. Many informational privacy concerns are about the internal workings of institutions managing our data, as opposed to external threats. People might worry that organisations do not understand the importance of their data, for example, and therefore will not take their informational privacy concerns seriously. They might worry that institutions will collect data unnecessarily—more, and more sorts of, personal information than they need, for example—or retain them unnecessarily, or that they will use the data in unscrupulous ways, such as to target vulnerable individuals for certain ends, or to use information for commercial purposes. Here, a trust-based strategy can clearly do some work. If the public trusts organisations responsible for public health information—because the public see those organisations as trustworthy on the basis of shared values—worries about the way those organisations process their data are dispelled. To take a simple example, people will not withhold information useful for public health purposes on the grounds that the relevant institutions will sell the data on, if they trust those institutions on the basis that commercialisation of personal data is at odds with the values they share with those institutions.

This evaluation of the trust-based strategy—i.e., trust deals well with privacy concerns about internal, but not external, threats—has been presented rather starkly, for the sake of clarity. A more nuanced account would address the way in which eliciting trust based on value congruence might subtly inform—if not comprise—organisations’ responses to external threats. After all, the way institutions go about ensuring reliability by better regulations varies. Some will take data protection more seriously than others; some will be profligate in their data security spending, some will protect certain sorts of data more than others, or apply different thresholds for when or how to respond to threats of data breaches, and so on. People can be reassured that institutions they trust on the basis of shared values will go about regulation in ways in which they approve; for example, they will take external threats seriously without wasting public money. In turn, this provides the public with further reassurance leading to increased willingness to share their data.

## Empirical Work

The main aim of this paper is theoretical—i.e., to clarify and defend the trust-based response to informational privacy concerns in public health—but we end by highlighting the need for further empirical work to build on these theoretical foundations. We need to know much more about public trust, perception of shared values, and barriers and willingness to contribute personal information for public health purposes. In this regard, there is a deep irony in the extant empirical data. On the one hand, there is important empirical work on trust, value congruence and willingness to contribute information, but it is not about health. On the other hand, there is important empirical work on trust, value congruence and health, but it is not about willingness to contribute information.

This irony is illustrated by two excellent empirical studies. In the first, Cazier et al. explore ‘how value congruence contributes to the formation of trust in e-businesses, and how trust and value congruence influence consumers to share personal information’ [2]. Their hypothesis was that the values of an organisation affect both trusting beliefs and willingness to share personal data; their method was to correlate 775 participants’ perceived value congruence with organizations, trusting beliefs, and types of information they would be willing to disclose; their results indicate that value congruence mediates trust and increases willingness to disclose personal information. This is precisely the sort of empirical study we recommend, but it is not focused on health, so it obviously needs to be transposed from Cazier et al.’s context of e-businesses to the sorts of organisations in which we are interested, namely, those responsible for managing public health data.^21^

The second empirical study, by Kehoe and Ponting [14], is in the health field, but it is not focused on information; rather, they are interested in health care reform and, in particular, ‘levels and determinants of trust in a health care system and in key actors in the health policy community’. So, whilst their analysis of determinants of public trust is based on value congruence and their results are promising—perceptions of shared values correlate to levels of trust—the focus of Kehoe and Ponting’s research is not quite right for the present context. And there are other idiosyncrasies of their study which would not be appropriate to our topic. In particular, in testing the relationship between value congruence and levels of trust, Kehoe and Ponting focus on just one specific value—namely, ‘accessibility’, understood as equal access to good quality health care, as found in the Canada Health Act—which serves as a proxy for shared values in general. So, as with Cazier et al.’s study [2], we would advocate taking the template Kehoe and Ponting provide, but applying it with suitable adjustments to the empirical studies of trust, shared values (broadly defined, not restricted to the one value they considered), and willingness to share personal information for public health purposes.

Although this is nascent, we can at least sketch some of the main items on this empirical research agenda. We need to know whether, how, and to what extent people trust institutions with their public health data; what are the main barriers to, and facilitators of, trust in those institutions—ensuring that value congruence is central in pursuing this—and what admixture of reliance and trust is most effective in reducing informational privacy concerns and increasing willingness to contribute data. This should assess people’s knowledge of how their health data are used, including the sorts of misunderstanding and misperception which preclude trust. ‘Determinants of trust’, an established subtheme in related research, is clearly key: what predisposes individuals to trust or mistrust public health institutions, and what shifts their predisposition from an attitude of mistrust to trust? Since public trust is grounded in shared values, the public’s understanding of the values espoused and embraced by public health institutions is clearly central. In particular, which values ground the public’s perception of public health institutions as trustworthy, and what can institutions responsible for managing public health data do to foster or lose the public’s trust?^22^

A final suggestion: we hypothesise that the sort of public health information in question will be a factor in determining the influence of trust, trustworthiness, and willingness to contribute personal data. In this respect, a taxonomy of public health information of the sort provided by Holland [11 at pp. 2–9] is key. For example, it seems likely that trust and value congruence are pretty much irrelevant to public health data collection mandated by legislation (for example, notifiable diseases), but they will have some influence on willingness to allow routine data collection (such as, ongoing health monitoring and surveillance systems), and will be highly influential in determining willingness to disclose personal information in unanticipated or unusual circumstances, such as public health emergencies, outbreaks and pandemics. This suggests that the requisite empirical work needs to be sufficiently fine-grained to be specific to sorts and categories of health information.

## Footnotes

1 Institutions responsible for collecting, processing, and using data for public health purposes include nationwide organisations such as the English National Health Service and Public Health England; regional institutions, for example, public health observatories; individual bodies responsible for specific sorts of public health data, such as biobanks; and research units which acquire and analyse information for public health purposes.

2 Obviously, not all data collection creates dilemmas, but our paper is motivated by the ever-present tension between gathering health data and privacy concerns, a tension which threatens to, and often does, create a utility-privacy dilemma.

3 At this stage, we put ‘trust’ in scare quotes when referring to the current strategy of building ‘trust’. As will come clear, this is to distinguish the concept of trust as it figures in this strategy from trust as it figures in the philosophical analysis, as discussed below.

4 By focusing solely on institutions which process public health data we avoid the ‘endless disputes over the definitions of key terms such as institution and organization’ and cognates such as ‘groups’ and ‘collectives' [9]. It is clear as to which institutions we are focused on—those responsible for acquiring and using public health information—so nothing hangs on whether we refer to these as institutions, organisation, etc., or on differences between these sorts of entities.

5 The philosophical literature on trust is vast, but as an indication, Baier [1] is a *locus classicus*; Jones [13] and Hardin [7] illustrate important refinements; and McLeod [17] provides an overview.

6 ‘Reactive attitudes’ was coined by Strawson [23] to refer to attitudes—such as, resentment, gratitude, moral indignation, love, and forgiveness—we take to one another in our practice of holding each other responsible. Although we borrow the term, Strawson’s project is to explain moral responsibility in a way that avoids a standoff between compatibilists/incompatibilists, which is clearly very distant from ours.

7 This is not to deny that parties on whom one relies can have motives and values which one recognises and even shares. A dentist might be motivated by concern for their patients and value healthy teeth, for example. But in the case of mere reliance, one’s perception of such motives and values goes into the calculation as to whether they are a reliable dentist. That’s why such motives and values are fungible: I might decide to rely on a particular dentist because I think they value me and my teeth, or because they value not getting sued or struck-off, or for quite different reasons. By contrast, in the trusting relationship, motives and values *ground* one’s perception of the trusted party as trustworthy.

8 For a list of detailed illustrations, see [11 at p. 117]. Although we are focused on public health information, this conflation of trust and reliance is ubiquitous in discussions of other (sometimes related) public health issues; for example, ‘Trust is a vital component of human interaction. When we *trust*, we *rely* on another to do the right thing …’ [5 at pp. 5–6; emphases added].

9 On a methodological note, there are important difference in approaches to these issues. On the (relatively few) occasions philosophers discuss public trust, they tend to ask very general questions about trust and institutions, such as, whether there is such a thing as organisational trust. For example, Townley and Garfield [25 at p. 96] ‘apply a distinction between trust and reliance to institutions’ and Hawley [8 at p. 232] examines ‘the costs of abandoning the trust-reliance distinction in collective contexts’. By contrast, like Sheehan et al. [21], we think it is more fruitful to examine trust and trustworthiness in very specific ‘institutions and collective contexts’: in our case, those responsible for public health data. After all, and as Hawley is aware, different accounts of trust emphasise different features—she gives the examples of ‘mutuality, emotional affect, and complex relationships’ [8 at p. 232]—so we need a more fine-grained analysis of how trust applies to specific sorts of institutions, not collectives in general.

10 Sheehan et al.’s dialectic [21] is similar to ours: using patient data from the National Health Service for research is often said to be a matter of trust; but documents, policies, guidelines etc., which emanate from this ‘are apparently constructed with little sustained reflection on the nature of trust and trustworthiness’; so they appeal to the philosophical distinction between trusting and relying or depending, arguing that while ‘philosophers have primarily thought about trust in the context of interpersonal relationships, these features of trust and trustworthiness … can help us think through their role in the context of using patient data for research in the NHS’ (pp 1–2). But our discussion also differs from Sheehan et al.’s in a number of respects. In particular, they are focused on NHS patient data for research so, for example, they are concerned with difficulties around trust and trustworthiness created by the complexity of the NHS. By contrast, we are interested in all forms of public health information (not just research data) and the whole nexus of institutions responsible for processing it (not just the NHS). More generally, we think there is more to be done to clarify, defend and critique the appeal to trust and trustworthiness in public health data management than Sheehan et al. imply.

11 The way in which institutions demonstrate values they share with the public is crucial. For example, commentators distinguish ways in which values are displayed: ‘Public institutions that are the repositories and beneficiaries of public trust make tacit or explicit commitments to uphold the requisite values’ [25 at p. 103]. But it is crucial to the strategy under discussion that institutions make explicit, not tacit, commitments to values, if they are to elicit public trust: the public will perceive institutions as trustworthy only if the latter’s values are clearly on display. The practicalities of this—i.e., how organisations responsible for public health data can most effectively display value alignment with the public—is clearly an important further research question that will centre on strategies for developing, refining, and publicising shared values.

12 Of course, even institutions which the public trusts on the basis of value congruence can end up behaving in harmful ways, for lots of reasons (e.g., unavoidable outside pressures). But trust can always be misplaced, and the likelihood of an institution letting the public down is surely much reduced when the institution shares the public’s values.

13 The ambiguity between trusting institutions and trusting the professionals who staff them runs through the literature on this topic. For example, Sheehan et al.’s [21] discussion segues from trust and trustworthiness of institutions responsible for patient data, into trust and trustworthiness of professionals, such as Caldicott Guardians and clinician researchers. We resist attempts to shift the focus from institutions to individuals: see, e.g., Hawley [8 at p. 246] who suggests that we ‘direct trust-related reactive attitudes towards groups … but could more appropriately direct these towards individuals, known or unknown, group members or not, who are in various ways responsible for the functioning of the group’. There is not space to detail the social ontology underpinning this—i.e., organisations must be something over-and-above the individuals involved in them— but for an indication of the position we favour in the debate between collectivists and individualists over the nature of organisations, see the ‘noneliminative individualism’ described by Cohen and Samp [3].

14 Of course, the degree of value congruence will vary between health systems. In this example, the distinctive history of the NHS, its place in British culture—including explicit reiterations of its underpinning values, and so on—play their part. But the fact that the idea of shared values sits less well with other sorts of health systems—insurance-based, for example—doesn’t undermine the point that value congruence can and in fact does exist between people and healthcare institutions.

15 For example, ‘the NHS and the research institutions that are associated with it should strive to be trustworthy and should be trustworthy. In general, thinking about the values associated with research in the NHS (like the direct relationship to patient benefit) and developing processes that reflect and secure these values, works towards trustworthiness’ [21 at p. 2].

16 This is only one of numerous such refinements on offer: e.g., Kehoe and Ponting [14] ‘identify the determinants of three different types of trust—generalized systemic trust, fiduciary trust, and generalized trust in particular actors’ input to health system changes’. We refer to this paper below as an important template for the kind of empirical work we envisage in this area.

17 This illustration is interpersonal (between the passenger and their taxi driver) so it seems to take us back to trust in individual professionals, as opposed to organisations, which, as just argued, is inappropriate in the public health context. But the idea here is to adapt the specific/general trust distinction to organisations.

18 To present a very straightforward illustration for the sake of clarity, imagine you have been swindled out of some money by your best friend, Save the Children Fund, or the local second-hand car dealership. How would you feel? On our account: horribly betrayed in the first case, somewhat betrayed in the second, and unsurprised in the third.

19 Obviously, this is not to deny that public health data collection creates privacy concerns—there would be no need to have this discussion if that were the case—and earlier we alluded to a case in point, namely, informational privacy concerns in the UK about the government’s Covid-19 ‘track and trace’ system [4]. Nonetheless, our point here is that mistrust of governments has variable significance in public health—particularly significant in the context of state action that is paternalistic as opposed to being aimed at avoiding third-party harms, for example—and its impact is not so strong as to nullify the strategy of augmenting reliance with trust in the context of information gathering. In addition, whether and to what extent mistrust of government is a barrier to the strategy under discussion is an independent datum in the empirical investigation we call for at the end of this paper: if it turns out to be a major barrier, so be it, but we hypothesise that it will not.

20 Sheehan et al. concur: ‘Particularly in cases in which consistent and guaranteed performance is required, we may be better off relying rather than trusting. For example, the data systems and infrastructure which house patient data should be as secure as possible as well as being as robust and well curated as possible. We want assurances that these systems function in a way that protects patient data from misuse, error, and corruption. Having such assurances is not a matter of trust or trustworthiness, but one of reliability’ [21 at pp. 2–3].

21 On a theoretical note, we would question the way Cazier et al. characterise the relationship between value congruence, trust, and willingness to contribute personal information [2]. They suggest that value congruence increases willingness to contribute personal information in two ways: indirectly, by increasing levels of trust which, in turn, increases willingness to disclose; and by directly increasing willingness to disclose. But the latter, direct, effect is theoretically dubious. If disclosing personal information is an issue at all (as opposed to divulging information in very intimate personal relationships, where it is not an issue, for example) it is an informational privacy issue; in which case, it is a trust issue. So, value congruence can only increase willingness to contribute personal information indirectly, i.e. by increasing levels of trust sufficient to overcome privacy concerns, and not, as Cazier et al. suggest, directly.

22 The aim is to elicit trust based on trustworthiness, so in pursuing this, empirical investigators will need to be alert to the distinction between trust and trustworthiness. This is important, because there is evidence of acquiring public trust, but on bases other than trustworthiness. For example, ‘public trust may be gained through public involvement, but the research institution may not be trustworthy’ [6 at p. 3].
